# Transcontinental Dispersal of Nonendemic Fungal Pathogens through Wooden Handicraft Imports

**DOI:** 10.1128/mbio.01075-22

**Published:** 2022-06-29

**Authors:** Jason A. Smith, Tania Quesada, Gideon Alake, Nicolas Anger

**Affiliations:** a School of Forest, Fisheries and Geomatics Sciences, University of Florida, Gainesville, Florida, USA; CDC

**Keywords:** environmental microbiology, fungal pathogens, invasive microorganisms, pathogens, plant pathogens

## Abstract

This study examined the viability and diversity of fungi harbored in imported wooden handicraft products sold in six retail stores in Florida, United States. Despite being subjected to trade regulations that require various sterilization/fumigation protocols, our study demonstrates high survival and diversity of fungi in wood products originating from at least seven countries on three continents. Among these fungi were nonendemic plant and human pathogens, as well as mycotoxin producers. Several products that are sold for use in food preparation and consumption harbored a novel (to North America) plant and human pathogen, *Paecilomyces formosus*. In addition, a high number of species isolated were thermophilic and included halophilic species, suggesting adaptability and selection through current wood treatment protocols that utilize heat and/or fumigation with methyl-bromide. This research suggests that current federal guidelines for imports of wooden goods are not sufficient to avoid the transit of potential live pathogens and demonstrates the need to increase safeguards at both points of origin and entry for biosecurity against introduction from invasive fungal species in wood products. Future import regulations should consider living fungi, their tolerance to extreme conditions, and their potential survival in solid substrates. Mitigation efforts may require additional steps such as more stringent fumigation and/or sterilization strategies and limiting use of wood that has not been processed to remove bark and decay.

## INTRODUCTION

International movement of microorganisms has garnered broad attention in recent years, primarily due to the threat from pathogens to the health of naive hosts (1–6). Fungal pathogens are increasingly recognized as a major risk and threaten agricultural and ecological systems worldwide (1, 3, 4, 6–9). The recent global emergence of several mycotic diseases is leading many public health experts to raise alarms about the need to address this new threat with more resources and research (1, 3–5, 8, 10–13). One important issue is uncertainty about sources of new strains that become established in vulnerable populations or in clinical settings from presumably natural/environmental inoculum reservoirs (1, 10–21). For example, fungicide-resistant strains are emerging in several species, including Candida auris (8), and Aspergillus fumigatus (22, 23), presumably due to widespread use of azole fungicides in agricultural and horticultural landscapes. Other examples include the emergence of virulent strains of Cryptococcus gattii affecting immunocompetent hosts and *Coccidioides* spp. that cause Valley Fever (13, 17).

Exotic fungal pathogens present a particular risk due to their rapid emergence, low resistance in host populations, and limited surveillance infrastructure for detection. Invasive fungal pathogens are linked to major ecological disasters, including near extinctions of forest tree species, such as the American chestnut due to *Cryphonectria parasitica* (24) and Florida torreya due to Fusarium *torreyae* (25). They have also caused a global decline of amphibian populations due to chytridiomycosis caused by *Batrachochytrium dendrobatidis* and *Batrachochytrium salamandrivorans* (2, 26). Furthermore, numerous fungi present serious threats to food security, such as wheat stem rust caused by *Puccinia graminis* race UG 99 (4, 5).

Increasing international trade, higher capacity of shipping vessels, greater reliance on nonlocal agriculture, and the movement of horticultural plants have all been identified as major drivers of this worldwide phenomenon (3, 4, 6). Although safeguards and regulations exist to prevent inadvertent introduction of microbes and insects in wood products (6), these precautions are only as good as the enforcement mechanisms in place at the origin of export and biosecurity measures at ports of entry. Analyses of the system have revealed novel pathways recently (6) with significant focus on exchange of numerous wood and plant-based products (6). Wood products have long been a risk for importation of new invasive pests and pathogens in relation to agriculture and forestry. Most risk assessments (and regulations) related to wood products concern timber and wood-boring insect pests that they may harbor (6). Indeed, the impact from these pests has been staggering and has led to widespread attention (1, 4, 6, 24). With the exception of fungi carried by bark and ambrosia beetles harbored in wood, little attention has been given to the broader risk from fungi associated with the diverse array of wooden products that are imported into the United States through trade.

**FIG 1:**
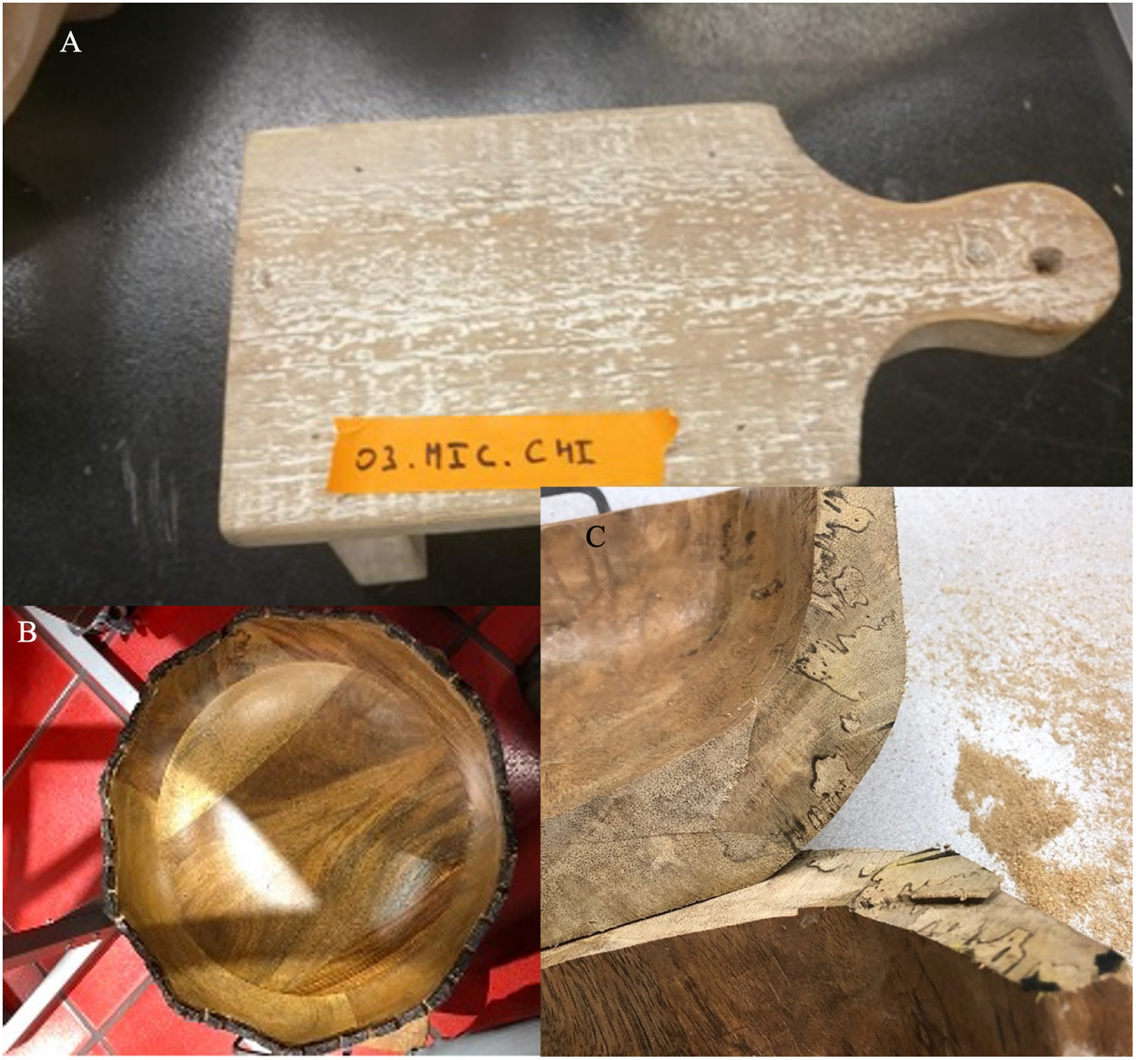
Three examples of wooden handicrafts tested in this study. (A) Wooden cutting board used in food preparation from China, with extensive white-pocket rot decay evident. (B) Bowl from Philippines used with bark present. (C) Bowl from Indonesia displaying decay and zone lines indicative of advanced fungal decay and colonization.

**FIG 2:**
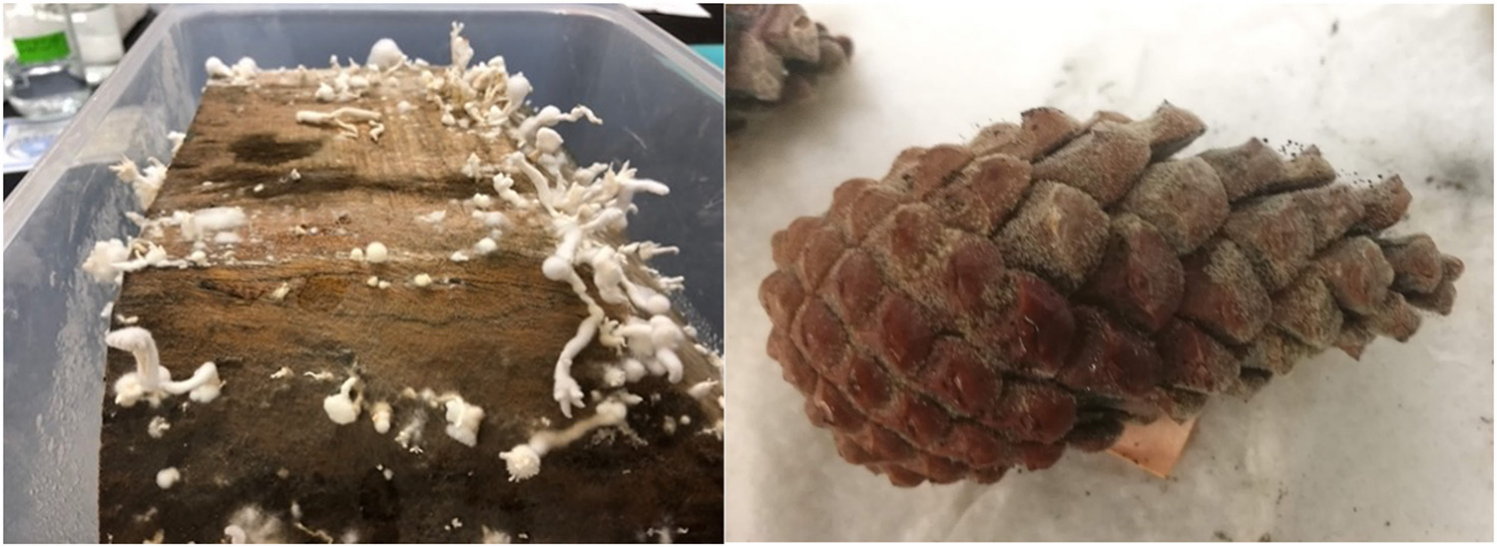
Samples following incubation in moisture chambers at 22°C. (Left) Wooden bowl from Philippines with immature fruiting bodies of *Schizophyllum commune*. (Right) Pinecone from Italy with multiple fungal fruiting bodies present.

**FIG 3:**
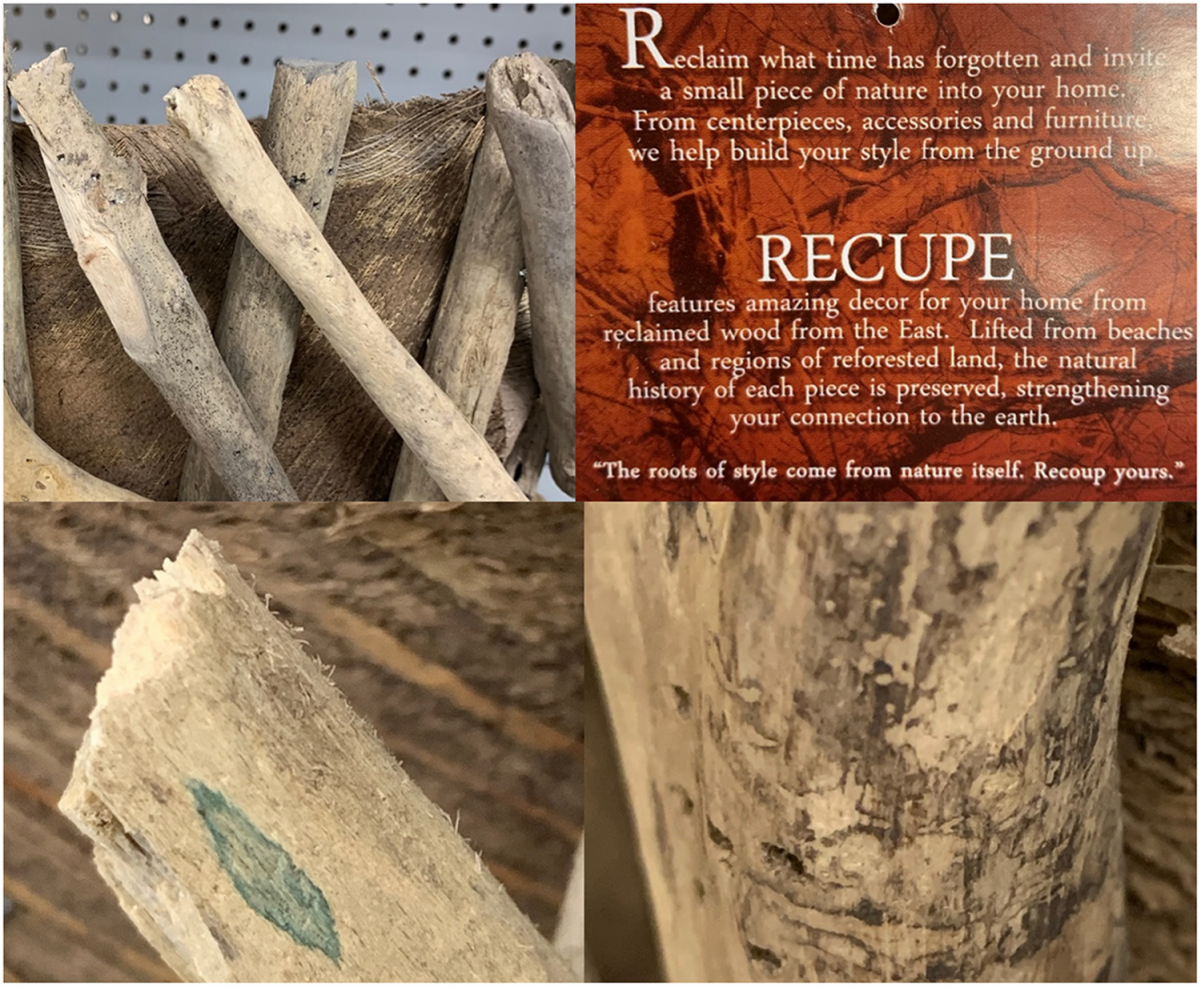
Decorative, mixed-materials bowl made of driftwood and coconut fiber sold in a retail store in Gainesville, FL (Origin: Philippines). Note the label in the upper right corner marketing “inviting nature into your home” using “reclaimed wood from the East.” Note also the green stain associated with recent fungal growth on wood in lower left panel and the zone lines and discoloration associated with recent decay fungi activity in lower right panel.

It was observed by the authors that many of the wooden home goods products (regulated by U.S. Department of Agriculture/Animal and Plant Health Inspection Service [USDA/APHIS] as “wooden handicrafts”) commonly sold at retail stores have foreign export origins, including many Asian countries, Europe, Mexico, and Central America. Furthermore, it was also observed that these products are not being produced from milled/processed wood but often made from weathered, decayed wood that contains bark (Fig. 1) or evidence of discoloration. For example, in the arts and crafts market, raw “driftwood,” pinecones and straw from foreign destinations is being sold (Fig. 2) and marketed in U.S. retail stores specifically due to their rustic, “straight from nature” appeal (Fig. 3). In some cases, evidence of microbial activity was observed on products, indicating potential reservoirs for organisms during importation (Fig. 3).

The current regulations from USDA/APHIS (27), encourage importers to engage with one of the 1,112 export firms in China (as of August 2021) that have been deemed to have met the APHIS standards. In short, this includes approved biosecurity facility, manufacturing process, and shipping requirements. If an importer desires to purchase products from an exporter not on the approved list, they will need to meet certain requirements to be added to the list, which include proper treatment of all wood craft materials larger than 1 cm in diameter, semifinished or finished with any wooden components that have not been machined. The treatments include heat (wood core temperature minimum of 60°C for 60 min), methyl bromide “for components with a diameter less than 15.24 cm/6 in. and larger than 1 cm in order to ensure the fumigant gets proper penetration,” or pressure treatment (varies by weight) (27). Apparently, products containing bark or rough portions are still subject to inspection specifically for “wood-boring insects,” but no mention is made of microorganisms.

For wooden handicrafts from destinations outside China, importers are directed to the general e-permits website (https://www.aphis.usda.gov/aphis/resources/permits), but no specific information is provided. Handicrafts from countries other than China do not have a specific regulatory control. According to APHIS (27), the material can be inspected for pests and then released as outlined in 7 CFR 319.40-9. If pests are detected during inspection, then quarantine actions will be taken. Thus, these materials are not subject to the requirements outlined above.

The following research was conducted to assess the potential risk of intercontinental importation of exotic fungal pathogens on wooden handicrafts purchased from six retail stores in Gainesville, FL. Two studies were conducted, with study 1 being carried out as a pilot study and included undergraduate students enrolled in a lab-based course and study 2 was a larger repetition of study 1.

## RESULTS

A total of 47 pure fungal colonies were recovered from 14 out of 16 household and wooden handicraft products tested in this study (Table 1). From Study 1, a total of nine separate fungal taxa were recovered from four of the five products tested (Tables 1 and 2). The largest number of taxa, five (Aspergillus versicolor, *Paecilomyces formosus*, *Penicillium alfredii*, *Peniophora albobadia*, and *Schizophyllum commune*), were recovered from a wooden bowl that originated in Indonesia. A second bowl tested, from the Philippines, as well as *Larix* sp. cones from China, yielded one fungal taxon each, *S. commune* and Alternaria alternata, respectively.

**TABLE 1:** Top BLASTn results for ITS rDNA region for fungal isolates recovered from studies 1 and 2^*a*^

Isolate code	Top BLASTn result	% identity	BLAST statistics		Length (bp)
			Query coverage	E value	
RS1-S1-01 (MN547364)*	*Schizophyllum commune* (MH307932)	100	100	0	600
RS1-S1-02 (MN547365)*	*Schizophyllum commune* (MT103532)	100	100	0	600
RS1-S1-03 (MN547366)*	*Paecilomyces formosus* (LC317710)	100	94	0	590
RS1-S1-04 (MN547367)*	*Schizophyllum commune* (MF476007)	100	100	0	650
RS1-S1-05 (MN547368)*	*Peniophora albobadia* (KU530154)	99.77	100	0	661
RS1-S1-06 (MN547369)*	Aspergillus versicolor (MH625700)	100	99	0	595
RS1-S1-07 (MN547370)*	*Penicillium alfredii* (MK450726)	98	97	0	776
RS1-S1-08 (MN547371)*	*Schizophyllum commune* (MH307932)	99.85	100	0	674
RS2-S1-01 (MN547372)*	Alternaria alternata (MT453271)	100	100	0	608
RS2-S2-01 (MN547383)	*Talaromyces rotundus* (EU497950)	99.52	99	0	623
RS3-S2-01 (MN547375)	*Aporospora terricola* (DQ865098)	96.28	91	0	554
RS3-S2-02 (MN547376)	*Rhizosphaera kalkhoffii* (JX981459)	100	99.83	0	621
RS3-S2-03 (MN547377)	*Rhizosphaera kalkhoffii* (JX981459)	100	99.84	0	621
RS3-S2-04 (MN547378)	*Periconia macrospinosa* (JX981482)	99.83	100	0	587
RS3-S2-05 (MN547380)	*Coprinellus radians* (MH855978)	100	100	0	631
RS3-S2-06 (MN547385)	*Rhizosphaera kalkhoffii* (JX981459)	100	100	0	605
RS3-S2-07 (MN547386)	*Dictyoarthrinium sacchari* (MT482325)	98.83	87	0	515
RS3-S2-08 (MN547387)	*Rhizosphaera kalkhoffii* (JX981459)	100	100	0	606
RS3-S2-09 (MN547388)	*Fimetariella rabenhorstii* (KX869958)	99.49	100	0	585
RS3-S2-10 (MN547389)	*Chaetomium cochlioides* (MT279444)	100	100	0	592
RS3-S2-11 (MN547391)	*Sarea resinae* (MT809245)	100	100	0	588
RS3-S2-12 (MN547392)	*Hormococcus conorum* (KF993412)	100	92	0	564
RS3-S2-13 (MN547393)	*Hormococcus conorum* (KF993412)	100	93	0	564
RS3-S2-14 (MN547394)	*Sarea difformis* (MH857896)	99.15	100	0	587
RS3-S2-15 (MN547395)	*Sarea difformis* (MH857896)	99.18	99	0	608
RS3-S2-16 (MN547396)	*Sarea difformis* (MH857896)	99	100	0	588
RS3-S2-17 (MN547397)	*Sarea difformis* (MH857896)	99	100	0	587
RS3-S2-18 (MN547398)	*Chaetomium globosum* (KX674657)	100	99	0	793
**RS3-S2-19** (MN547400)	*Trichoderma longibrachiatum* (MT634694)	100	100	0	450
**RS3-S2-20** (MN547401)	*Curvularia* sp. (MT066189)	99.83	99	0	583
**RS3-S2-21** (MN547402)	*Curvularia lunata* (MT683262)	100	100	0	587
**RS3-S2-22** (MN547403)	*Trichoderma longibrachiatum* (KY225659)	100	99.81	0	533
**RS3-S2-23** (MN547404)	*Bipolaris austrostipae* (NR147491)	99.68	99	0	624
**RS3-S2-24** (MN547406)	*Trichoderma citrinoverde* (MG972800)	100	100	0	630
**RS3-S2-25** (MN547407)	*Rhizopus oryzae* (MT316366)	100	100	0	655
**RS3-S2-26** (OM262198)	*Schizophyllum commune* (MH307932)	99.84	100	0	645
**RS3-S2-27** (MN547409)	Paecilomyces variotii (FJ345354)	100	100	0	622
**RS3-S2-28** (MN534797)	Paecilomyces variotii (FJ345354)	100	100	0	447
RS4-S2-01 (MN547373)	Aspergillus flavus (CP051065)	100	100	0	636
RS4-S2-02 (MN547374)	*Xylaria badia* (GU322446)	98.44	95	0	586
RS4-S2-03 (MN547379)	*Coprinellus radians* (LC612525)	100	100	0	697
RS4-S2-04 (MN547384)	*Rhizopus delemar* (LC514308)	99.69	100	0	645
**RS4-S2-05** (MN547405)	*Rhizopus delemar* (LC514308)	99.84	99	0	646
RS5-S2-01 (MN547381)	*Coprinellus xanthothrix* (MK573918)	100	100	0	706
RS5-S2-02 (MN547382)	*Humicola grisea* (KU705826)	100	99	0	578
RS5-S2-03 (MN547390)	Aspergillus *pseudoglaucus* (MT316341)	100	100	0	569
RS5-S2-04 (MN547399)	Cladosporium cladosporioides (MT598826)	100	100	0	539

a *, Samples from study 1. GenBank accession numbers are in parentheses. Isolate codes indicated in boldface were recovered from samples incubated in moist chambers.

**TABLE 2:** Isolation of fungi in studies 1 and 2

Retail store	Product description	Country of origin	No. of fungal taxa recovered	No. of samples	Plating method
Study 1					
1	Wooden bowl^*a*^	Indonesia	5	9	Streaking
	Wooden coaster	India	0	1	Streaking
	Wooden bowl^*b*^	Philippines	2	9	Streaking
2	*Larix* sp. cones for crafts	China	1	5	Streaking
	Wooden eggs	China	1	1	Streaking
Study 2					
1	Dried grape vines 1	China	2	5	Streaking
	Dried grape vines 2	China	3	1	Direct plating
	Bread board platform^*c*^	China	1	1	Direct plating
	*Pinus* sp. cones^*d*^	Italy	11	1	Streaking
	Straw	Mexico	1	5	Streaking
	Wooden letter “S”	China	0	1	Direct plating
2	Wooden rice bowls/spoons	Thailand	2	1	Streaking
	Wooden spatula	China	0	7	Streaking
	Wooden serving tray^*e*^	Thailand	7	1	Streaking
3	Wooden “cookie” disks^*f*^	China	5	3	Streaking
4	Wooden sticks/driftwood	Philippines	2	9	Streaking

a Evidence of decay was present.

b Evidence of both decay and fungal zone lines were present.

c White pocket rot evident.

d Pycnidia present on cone scales.

e Bark and some sapstain present.

f Bark present. The number of samples refers to the number of times a tissue was collected for plating. Each sample was replicated twice per media type.

A total of 24 taxa were further recovered from 11 products purchased from four retail stores in study 2 (Tables 1 and 2). Eleven taxa, the largest number recovered from a single product, were obtained from *Pinus* sp. cones imported from Italy (*Aporospora terricola*, *Chaetomium cochlioides*, *Coprinellus radians*, *Dictyoarthrinium sacchari*, *Fimetariella rabenhorstii*, *Hormococcus conorum*, *Periconia macrospinosa*, *Rhizopus oryzae*, *Rhizosphaera kalkhoffii*, *Sarea resinae*, and *Trichoderma citrinoverde*), followed by four taxa from a wooden food serving tray from Thailand (Aspergillus flavus, *C. radians*, *R. delemar*, and *Xylaria badia*). The remainder of the taxa were isolated from grape vines from China sample 1 (*T. longibrachiatum*), grape vines from China sample 2 (*Bipolaris austrostipae*, *Curvularia lunata*, and *T. longibrachiatum*), a bread cutting board/serving tray from China (Paecilomyces variotii), straw from Mexico (*S. commune*), wooden sticks/driftwood from the Philippines (*Talaromyces rotundus*), and wooden coasters from China (*A. pseudoglaucous*, Cladosporium cladosporioides, *C. xanthothrix*, and *Humicola grisea*).

### Functional group assignment.

**FIG 4:**
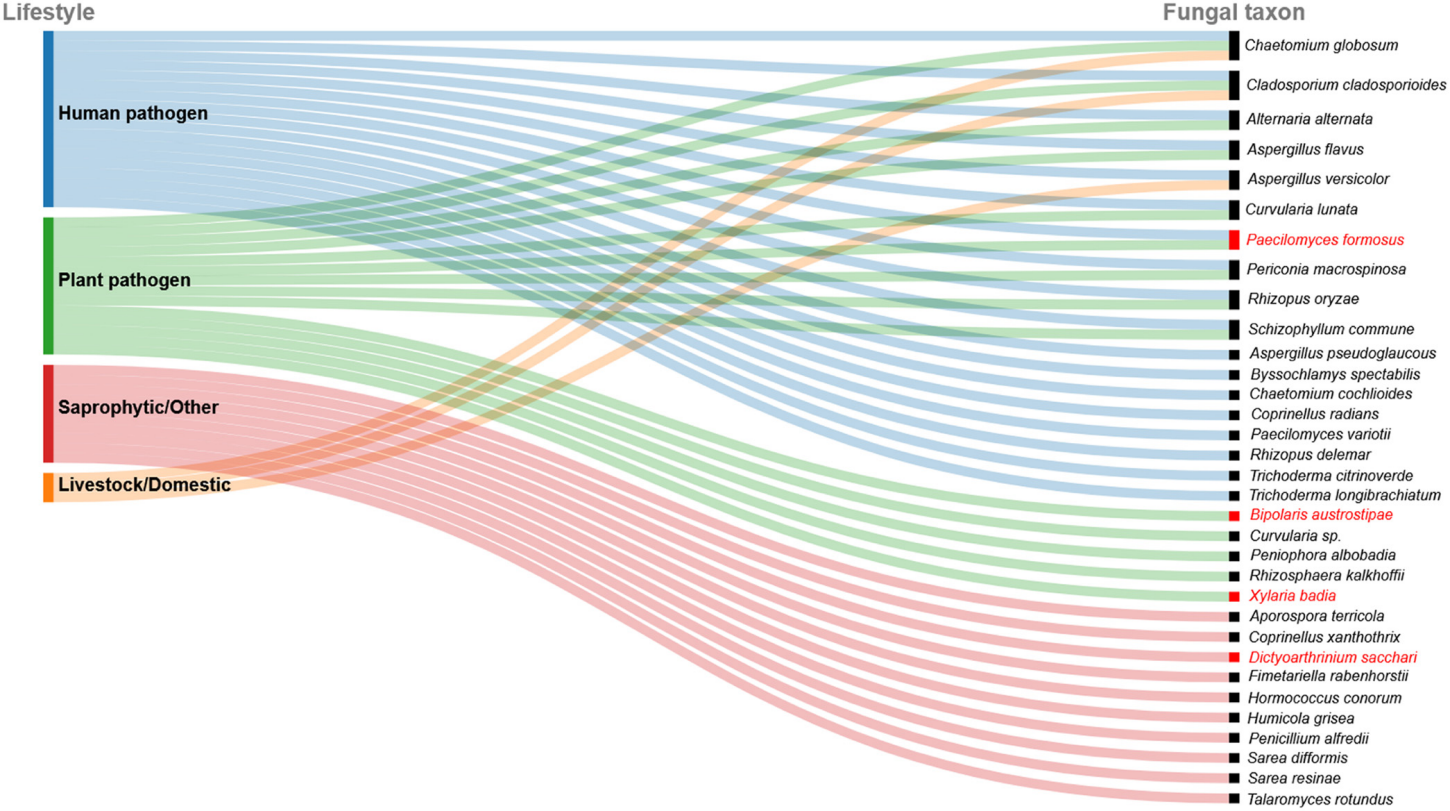
Fungal taxa organized by lifestyle/guild based on searches of multiple databases and literature searches. Several taxa are represented in more than one category. Taxa in red font are not known to have been documented in North America previously.

Of the 43 fungal taxa recovered from the two studies (Tables 1 and 2), the following 14 were determined to be plant pathogens (Fig. 4) (A. alternata (28), A. flavus (postharvest) (29, 30), *B. austrostipae* (31), *Chaetomium globosum* (32), *C. cladosporioides* (33), *C. lunata* (34), *Curvularia* sp. (34), *P. formosus* (35, 36), *P. albobadia* (37), *Periconia macrospinosa* (38), *R. oryzae* (39), *R. kalkhoffii* (40), *S. commune* (40), and *X. badia* (decay fungus) (41, 42), with three of these not previously reported in the United States: *B. austrostipae*, *P. formosus*, and *X. badia*. These pathogens represent disease agents of cereal crops, fruits/vegetables, trees, and postharvest pathogens, as well as decayers (29, 33, 40, 42). They also include several major mycotoxin producers, including A. alternata (28, 43), A. flavus (29, 30), A. versicolor (15), and *P. macrospinosa* (44). One saprophytic fungus, *D. sacchari*, has never been reported in the United States before (45; https://www.gbif.org), being found in a wide range of African, Asian, and Caribbean locations.

In addition, we recovered 17 taxa that are considered human pathogens (Fig. 4); these 17 represent over a third of all taxa recovered. Of these, *P. formosus* (18, 46), which is known from the Middle East and Asia, has not yet been reported in the United States. The others—A. alternata (28, 47–49), A. flavus (23, 50), *A. pseudoglaucous* (23, 51), A. versicolor (14, 15), *C. cochlioides* and *C. globosum* (49, 52, 53), *C. cladosporioides* (49, 54), *C. radians* (55), *C. lunata* (49, 56), P. variotii (9, 16, 21), *P. macrospinosa* (49, 57), *R. delemar* (58–60) and *R. oryzae* (61–64), *S. commune* (65), *T. citrinoverde* (66, 67), and *T. longibrachiatum* (66, 67)—are associated with clinical infection records at various global locations, including the United States.

Three livestock/domestic animal pathogens were also recovered (Fig. 4): A. versicolor (14), *C. globosum* (68), and *C. cladosporioides* (54, 69). These are also considered human pathogens.

**FIG 5:**
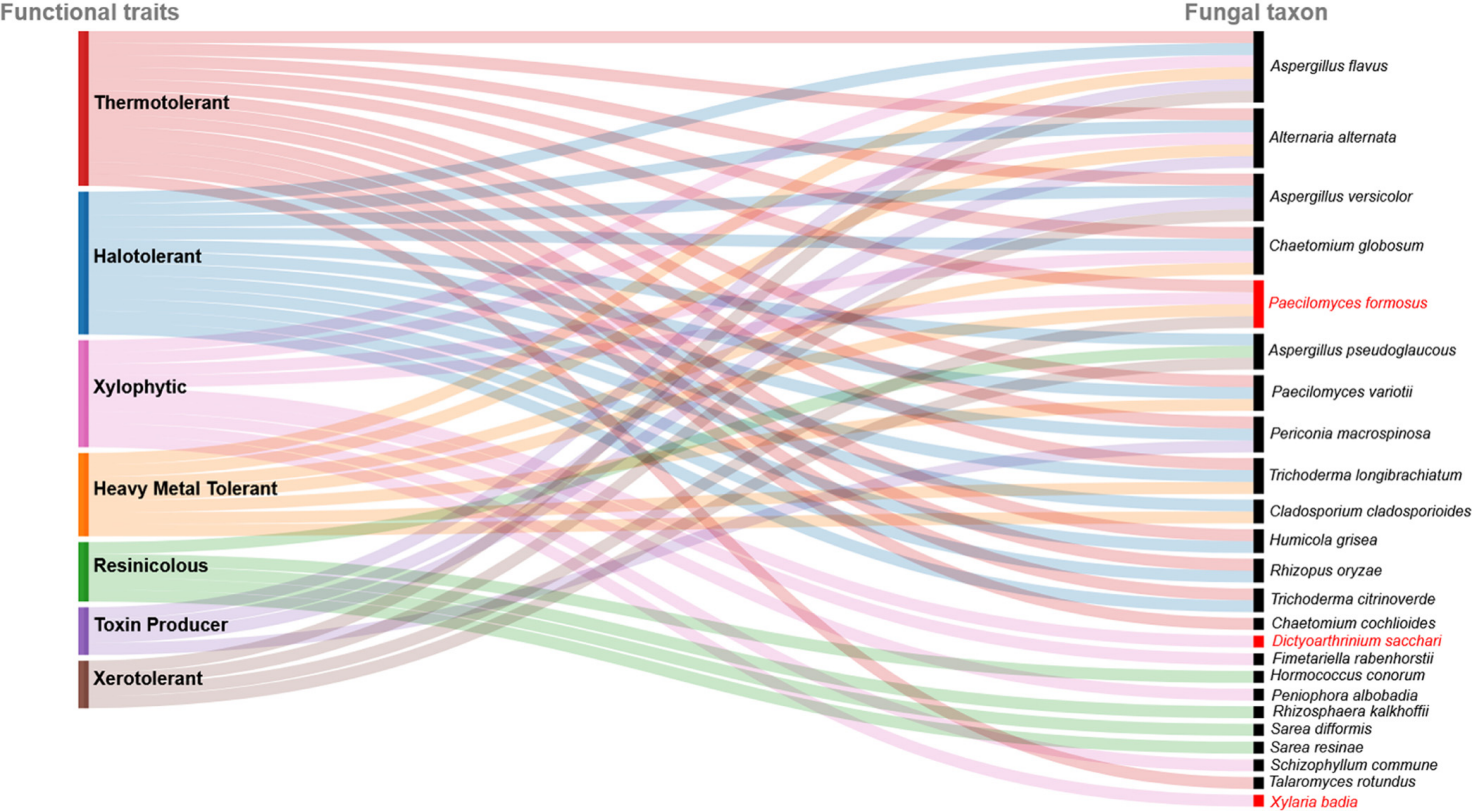
Fungal taxa organized by functional trait, based on searches of multiple databases and literature searches. Several taxa are represented in more than one category. Taxa in red font are not known to have been documented in North America previously.

Thirteen thermophilic/thermotolerant species were found and included A. alternata (28, 70, 71), A. flavus (70–73), A. versicolor (15, 51, 74), *Chaetomium* spp. (74, 75), *C. cladosporioides* (71, 74, 76, 77), *H. grisea* (74, 78), P. variotii (21), *P. macrospinosa* (44, 57), *R. oryzae* (79, 80), *T. rotundus* (81), *T. citrinoverde* (78), and *T. longibrachiatum* (78, 80). Many of these species are also halophilic/halotolerant, such as A. alternata (18, 70), A. flavus (29, 73), A. versicolor (73), *C. globosum* (70, 75), *C. cladosporioides* (76), *Humicola grisea* (78), P. variotii (21, 82), *P. macrospinosa* (44), *R. oryzae* (70, 79), *T. citrinoverde* (70, 83, 84), and *T. longibrachiatum* (70, 83, 84). Four xerophylic/xerolerant species were also found: A. flavus (29, 50, 51), *A. pseudoglaucous* (51), A. versicolor (15), and *P. formosus* (85). Five species were determined to be resinicolous based on the literature (*A. pseudoglaucous* [51], *H. conorum* [86–88], *R. kalkhoffii* [40, 86–88], and *S. difformis* [87, 88]), and nine species were identified as xylophytic (A. alternata [28], A. flavus [29], *C. globosum* [75, 89], *F. rabenhorstii* [90, 91], *P. formosus* [35, 36], *P. albobadia* [37], *S. commune* [40], and *X. badia* [decay fungus] [41, 42]). Other species also possess heavy metal tolerance; these species include A. alternata (92), A. flavus (50, 93), *C. globosum* (75), *C. cladosporioides* (76), *P. formosus* (85), P. variotii (21), and *T. longibrachiatum* (49, 93) (Fig. 5).

## DISCUSSION

This is the first known study to examine the viability of fungal pathogens in wooden handicrafts imported into the United States. These products are highly varied in their source materials, origin, destination, function/use, and biosecurity treatment prior to shipment. Although APHIS has rules that regulate the importation of these products, these regulations are based on potential risks to plant commodities based on products used in gardens (for example, bamboo stakes used in gardens). Little to no consideration appears to be given to products used in crafts or in food preparation (27). Furthermore, these rules were mainly written with potential wood-boring insects being prioritized as the greatest threat. Given that many of these products are destined for use in food preparation and/or utensils, handcrafting, or in bath and beauty industries, the potential threat of microbes to other hosts (nonplant; i.e., human) from this commodity appears to have been given little attention in the current regulations. Similar products exist in pet stores (toys, perches, ornaments, etc.). The studies reported here demonstrated that from a small sampling of products and retail stores, potentially dangerous and nonendemic fungal pathogens could be recovered.

The fungi recovered include 25 plant and human pathogens, including three that have never been reported in North America previously (Fig. 4): *B. austrostipae*, *P. formosus*, and *X. badia*. Although the exact distributions of these species are not clear, the latter two have been found previously in Asia (including China, the products’ import origin location), and in particular, on wood substrates. *Bipolaris austrostipae* has only been found in Australia previously, but all three of these are plant pathogens, and *P. formosus* is both a plant and human pathogen. *Bipolaris austrostipae* was described from Queensland (31), Australia, on *Austrostipa* sp., and other species of *Bipolaris* are important plant pathogens on numerous hosts globally. Several are important emerging fungal pathogens, and some species (31) are known as human pathogens (49). The global importance of *B. oryzae* as a major pathogen of rice cannot be understated as the cause of brown spot. This disease is known to have been a major cause of the Great Bengal Famine of 1942 when rice yield losses of 50 to 90% were reported due to an epidemic of this disease (94). *Paecilomyces formosus* is particularly interesting because it has the potential to infect both plants and humans (18, 35, 36, 46). It is associated with cankers and dieback of oaks and pistachio trees in the Mediterranean region and has also caused cutaneous and lung infections in both premature infants and immunocompromised adults in the Middle East (18, 46). It is not known whether strain-level differentiation exists for host specificity within this species but, given the wide potential for threats to both tree and human hosts, this nonendemic species is of significant concern and should be a surveillance target in future sampling. Interestingly, *X. badia*, thought to function as a decay fungus of wood, was recently described (42) from the Mekong Delta region of Vietnam, where it is apparently endemic. The only other record was as an endophyte of orchids in tropical regions of China (41). This species was recovered from a product imported from China; however, the exact origin of the original raw wood material is unknown.

Many wooden handicraft commodities are made up of minimally or unprocessed wood. The widespread popularity of “rustic” home goods products—those with bark, decay, zone lines, and discoloration present—increases the likelihood of fungi being present. Likewise, products used in arts and crafts, such as pinecones, grape vines, and driftwood, are unprocessed and raw material and likely to contain a wide diversity of microbial organisms. Indeed, in this study we recovered fungi from products containing unprocessed wood and products containing bark and evidence of decay, suggesting fungi were already well established when shipped.

An evaluation of the functional traits (Fig. 5) of the fungi recovered illustrates a high proportion of the taxa (76.6%) with tolerances that would increase their likelihood for survival through potential heat and/or methyl bromide treatments (thermophilic/thermotolerant, halophilic/halotolerant, xerophylic/xerotolerant, xylophytic, resinicolous, and heavy metal tolerant). Although it is difficult to determine the exact treatments for these samples, knowledge of the fungi recovered and their tolerances can guide new strategies for policies to reduce future introductions and develop new treatment methods that can control the viability of fungi within imported wooden products.

These results suggest that the current regulations are not sufficient to prevent entry of novel fungal pathogens on wooden handicrafts. Although numerous plant and human pathogens were recovered from this study, the significance of the results should be placed on the overall potential pathway rather than on individual species and illustrate the importance of including fungi in risk assessments associated with wood-based commodities.

Future strategies for mitigating risks associated with importation of novel fungal pathogens on wooden handicrafts could be employed by increasing stringency standards for the products themselves and the treatment process. The importation of rustic, unprocessed wood with bark and/or decay and of products such as cones, straw, and driftwood would likely increase the risk of entry of microbes associated with these products. Although our study did not evaluate this variable, we did observe numerous products with evidence of fungal growth (staining, fruiting bodies, zone lines). By limiting imports to clean, processed wood only, the probability of importing established fungal growth is reduced. Likewise, by standardizing the product raw materials, fumigation and other sterilization treatments can be improved and designed to match the substrate and adequately sterilize taking into account potential thermophilic and halophilic taxa as identified in this study.

## MATERIALS AND METHODS

Two studies were completed during 2019 and 2020 to examine the survival and diversity of fungi in wood products sold in retail stores in Gainesville, FL.

Undergraduate students in a forest pathology course participated in the processing and culturing of samples purchased by the first author at two retail stores. This was considered a pilot study that is described as study 1. Five products (Table 2) were purchased from two retail stores, representing four countries of origin, transported to the laboratory and maintained at 4°C until processed within 3 days of purchasing. Processing involved culturing on selective media (potato dextrose agar [Gibco] acidified with 1 mL/L lactic acid [APDA], malt extract agar [MEA; Gibco], and basidiomycete select agar [BSA]), and incubation of select wood pieces in moist chambers. Fungal cultures were identified by Sanger sequencing of the internal transcribed spacer region (ITS) rDNA, followed by sequence comparisons using BLASTn searches in NCBI (https://blast.ncbi.nlm.nih.gov/Blast.cgi) (Table 1).

In study 2, a larger experiment was completed with 11 products sampled from four additional retail stores, representing five countries of origin (Table 2). The products were handled in the same way as study 1 and were also processed within 3 days of purchasing. Processing involved culturing and sequencing cultures as described above.

### Wood product sampling.

The objects (Fig. 1) were surface sterilized with 70% ethanol to remove any external material and were drilled with an oscillator saw or drill. Cutting instrument parts that touched the wood, such as blades, and drill bits, were surface sterilized with 96% ethanol and flamed between each sampling. Sawdust or wood/straw pieces were obtained from the samples. Samples varied in size and some included multiple components such as several utensils packaged together. Sampling was done to ensure all components that were packaged in aggregate were sampled (Table 2). Between one to nine samples were taken from the products using the cutting instruments (Table 2). Two replicates from each sample were then plated onto one plate each for each replicate and medium type. In the case of sawdust, approximately 25 mg for bark and 100 mg for cambium and other tissue types was resuspended in 500 μL of sterile double-deionized water. Sawdust samples were aliquoted (100 μL), vortexed and then streaked with a sterile glass rod onto plates of three different types of media MEA, APDA, and BSA. Wood or straw pieces, approximately 50 mm × 10 mm in size were excised from the larger sample using a sterile scalpel. These were sterilized further with half-strength bleach (5% sodium hypochlorite) diluted with sterile water for 30 s, followed by a wash in sterile autoclaved water prior to plating four times (pseudoreplicates) onto the three respective medium types in a sterile laminar flow hood. Plates were wrapped in Parafilm (Bemis, Inc.) and then incubated at room temperature in the dark and monitored daily for fungal growth. The specific culturing methods applied for each sample are available in Table 2.

### Wood tissue incubation.

To encourage the growth of existing viable fungi present inside wooden handicrafts, products cut in half (study 1) or wood pieces (study 2) were incubated in moist chambers. Two wooden bowls obtained from the Philippines and Indonesia and wooden coasters from China exhibiting evidence of fungal colonization were used for incubation in study 1. The products were surfaced disinfected with 10% sodium hypochlorite and then cut in half with a sterilized cutting tool. The wood pieces were soaked in sterile water for 1 h, and then two replicates were placed in sterile plastic boxes lined with premoistened sterile paper towels. The boxes were sealed with Parafilm and maintained at room temperature and monitored every 1 to 3 days for emergence of fungal fruiting bodies. No fungal fruiting bodies in study 1 were used for sequencing. Six products (derived from samples 01-MIC-CHI, 02-MIC-CHI, 09-ROS-THA, 04-MIC-ITA, 05-MIC-MEX, and 03-MIC-CHI, isolates highlighted in boldface in Table 1) were used for incubation in study 2. Cross-sections of wood, 2 to 4 cm in diameter (or straw for 05-MIC-MEX, 2 cm in length), were surface sterilized by washing with 5% sodium hypochlorite, allowed to dry, followed by spraying with 70% ethanol. Two (for wooden products) or six (straw products) replicates were then placed in sterile plastic boxes lined with premoistened sterile paper towels. The boxes were sealed with Parafilm and maintained at room temperature and monitored every 1 to 3 days for the emergence of fungal fruiting bodies. In addition, cones from an unidentified *Pinus* sp. imported from Italy were incubated the same way; however, these were maintained whole, rather than cut, with six replicates being used. Fungal growth was identified by scraping fruiting bodies that developed and identification by DNA extraction and Sanger sequencing of the ITS rDNA as described above (Table 1).

### Fungal culture identification.

Within 24 to 72 h, individual colonies were transferred to new plates when they were observed to be culturable (free of contamination by neighboring microbial growth). Colony counts were not completed, and growth was not quantified for this study because the growth was generally too heterogenous and in many cases groups of colonies were consolidated, making accurate quantification of growth impossible. Isolates were subcultured onto the same type of media they were originally isolated from by aseptically transferring a small plug of mycelia from the edge of the colonies. Isolates were grouped into unique morphotypes and given accession codes. If possible, unique morphotypes were counted for each product tested and, in several cases, multiple products produced the same morphotype. Once colony growth reached approximately 2.5 cm in diameter, a sample of mycelium was scraped off and used for DNA extraction with Extract-N-Amp procedure (Sigma). PCR amplification was performed using the primers ITS1F-ITS4 (95). PCR amplicons were visualized on a 1.2% agarose gel stained with SYBR green (Sigma-Aldrich, Inc., St. Louis, MO) to confirm amplification prior to Sanger sequencing. Sequences were aligned using Geneious 10.2.6 (http://www.geneious.com) software, and low-quality bases were trimmed and/or edited to produce a final consensus sequence for tentative identification. Final aligned sequences were deposited in GenBank (Table 1).

Sequences were compared against the NCBI database using BLASTn (https://blast.ncbi.nlm.nih.gov/Blast.cgi), using default settings to determine the potential taxonomic identity of the isolates (Table 1). BLAST results were considered a “top match” based on the following criteria: (i) high identities/query coverage (query coverage of > 95% was cutoff); (ii) matching well-annotated specimens, with priority given to deposited sequences originating from living cultures, particularly vouchers; and (iii) sequences deposited from noncultured sources (i.e., metasequencing studies) were filtered and not considered in the analyses. If identifications could not be determined (identity 90 to 95%) at the species level, but there was no ambiguity at the genus level, the identification was designated as “Genus sp.” No identifications were given for identities below 90%.

### Fungal biogeography and functional group determination.

Each fungal taxon name was checked against Index Fungorum (www.indexfungorum.org) and MycoBank (https://www.mycobank.org) for current taxonomic status and synonyms. For each of the taxa, the biogeography was assessed using multiple sources, including both Index Fungorum and Mycobank, as well as the U.S. National Fungus Collections Nomenclature Database (https://nt.ars-grin.gov), the National Center for Biotechnology Information life-map tree database (http://lifemap-ncbi.univ-lyon1.fr/), and the Global Biodiversity Information Facility (GBIF; https://www.gbif.org) using the “occurrences” filter to determine known records for the taxa identified in this study. For each of the taxa searched, their biogeography, lifestyle (pathogen [and type] versus saprophyte) and toxin production ability was determined by searching the literature using multiple databases, including PubMed (https://pubmed.ncbi.nlm.nih.gov), Google Scholar (https://scholar.google.com), Agris (https://www.fao.org/agris/), MycoPortal (https://mycoportal.org/portal/), and GBIF (https://www.gbif.org). In addition, they were grouped based on traits determined to increase likelihood of survival through the biosecurity measures: halophile, resinicolous, thermophile, xerophile, and xylophyte. Tolerance to heavy metals was also determined based on the literature. Data for each analysis was visualized (Fig. 4 and 5) using Raw Graphs (https://rawgraphs.io) and then figures were exported, and text and colors were customized using an html editor.

## References

[1] Anaissie EJ, Bodey GP, Rinaldi MG. 1989. Emerging fungal pathogens. Eur J Clin Microbiol Infect Dis 8:323–330. doi:10.1007/BF01963467. 2497012

[2] Fisher MC, Garner TWJ. 2020. Chytrid fungi and global amphibian declines. Nat Rev Microbiol 18:332–343. doi:10.1038/s41579-020-0335-x. 32099078

[3] Fisher MC, Gurr SJ, Cuomo CA, Blehert DS, Jin H, Stukenbrock EH, Stajich JE, Kahmann R, Boone C, Denning DW, Gow NAR, Klein BS, Kronstad JW, Sheppard DC, Taylor JW, Wright GD, Heitman J, Casadevall A, Cowen LE. 2020. Threats posed by the fungal kingdom to humans, wildlife, and agriculture. mBio 11:e00449-20. doi:10.1128/mBio.00449-20. 32371596 PMC7403777

[4] Fisher MC, Henk DA, Briggs CJ, Brownstein JS, Madoff LC, McCraw SL, Gurr SJ. 2012. Emerging fungal threats to animal, plant, and ecosystem health. Nature 484:186–194. doi:10.1038/nature10947. 22498624 PMC3821985

[5] Fones HN, Bebber DP, Chaloner TM, Kay WT, Steinberg G, Gurr SJ. 2020. Threats to global food security from emerging fungal and oomycete crop pathogens. Nat Food 1:332–342. doi:10.1038/s43016-020-0075-0. 37128085

[6] Linnakoski R, Forbes KM. 2019. Pathogens: the hidden face of forest invasions by wood-boring insect pests. Front Plant Sci 10:90. doi:10.3389/fpls.2019.00090. 30804966 PMC6378281

[7] Soare AY, Watkins TN, Bruno VM. 2020. Understanding mucormycoses in the age of “omics. Front Genet 11:699. doi:10.3389/fgene.2020.00699. 32695145 PMC7339291

[8] Spivak ES, Hanson KE. 2018. *Candida auris*: an emerging fungal pathogen. J Clin Microbiol 56:e01588-17. doi:10.1128/JCM.01588-17. 29167291 PMC5786713

[9] Steiner B, Aquino VR, Paz AA, Silla LMdR, Zavascki A, Goldani LZ. 2013. *Paecilomyces variotii* as an emergent pathogenic agent of pneumonia. Case Rep Infect Dis 2013:273848. doi:10.1155/2013/273848. 23819077 PMC3683431

[10] Bouza E, Muñoz P, Guinea J. 2006. Mucormycosis: an emerging disease? Clin Microbiol Infect 12:7–23. doi:10.1111/j.1469-0691.2006.01604.x.

[11] Chen J, Varma A, Diaz MR, Litvintseva AP, Wollenberg KK, Kwon-Chung KJ. 2008. *Cryptococcus neoformans* strains and infection in apparently immunocompetent patients, China. Emerg Infect Dis 14:755–762. doi:10.3201/eid1405.071312. 18439357 PMC2600263

[12] Garg D, Muthu V, Sehgal IS, Ramachandran R, Kaur H, Bhalla A, Puri GD, Chakrabarti A, Agarwal R. 2021. Coronavirus disease (Covid-19) associated mucormycosis (CAM): case report and systematic review of literature. Pathologia 186:289–298. doi:10.1007/s11046-021-00528-2. PMC7862973 33544266

[13] McCotter OZ, Benedict K, Engelthaler DM, Komatsu K, Lucas KD, Mohle-Boetani JC, Oltean H, Vugia D, Chiller TM, Sondermeyer Cooksey GL, Nguyen A, Roe CC, Wheeler C, Sunenshine R. 2019. Update on the epidemiology of coccidioidomycosis in the United States. Med Mycol 57:S30–S40. doi:10.1093/mmy/myy095. 30690599 PMC6823633

[14] Charles MP, Noyal MJ, Easow JM, M R. 2011. Invasive pulmonary aspergillosis caused by *Aspergillus versicolor* in a patient on mechanical ventilation. Australas Med J 4:632–634. doi:10.4066/AMJ.2011.905. 23386878 PMC3562921

[15] Engelhart S, Loock A, Skutlarek D, Sagunski H, Lommel A, FäRber H, Exner M. 2002. Occurrence of toxigenic *Aspergillus versicolor* isolates and sterigmatocystin in carpet dust from damp indoor environments. Appl Environ Microbiol 68:3886–3890. doi:10.1128/AEM.68.8.3886-3890.2002. 12147486 PMC124040

[16] Houbraken J, Verweij PE, Rijs AJMM, Borman AM, Samson RA. 2010. Identification of *Paecilomyces variotii* in clinical samples and settings. J Clin Microbiol 48:2754–2761. doi:10.1128/JCM.00764-10. 20519470 PMC2916617

[17] Kirkland TN, Fierer J. 1996. Coccidioidomycosis: a reemerging infectious disease. Emerg Infect Dis 2:192–199. doi:10.3201/eid0203.960305. 8903229 PMC2626789

[18] Kuboi T, Okazaki K, Inotani M, Sugino M, Sadamura T, Nakano A, Kobayashi S, Ota A, Nishimura K, Yaguchi T. 2016. A case of cutaneous *Paecilomyces formosus* infection in an extremely premature infant. J Infect Chemother 22:339–341. doi:10.1016/j.jiac.2015.12.003. 26774294

[19] Litvintseva AP, Mitchell TG. 2012. Population genetic analyses reveal the African origin and strain variation of *Cryptococcus neoformans* var. *grubii*. PLoS Pathog 8:e1002495. doi:10.1371/journal.ppat.1002495. 22383873 PMC3285590

[20] Sasinuch R, Mejia-Chew C, Ayres C, Spec A. 2021. A canker barking at the wrong knee: *Thyronectria austroamericana* septic arthritis. Open Forum Infect Dis 8:ofab381. doi:10.1093/ofid/ofab381. 34458393 PMC8387459

[21] Urquhart AS, Mondo SJ, Mäkelä MR, Hane JK, Wiebenga A, He G, Mihaltcheva S, Pangilinan J, Lipzen A, Barry K, de Vries RP, Grigoriev IV, Idnurm A. 2018. Genomic and genetic insights into a cosmopolitan fungus, *Paecilomyces variotii* (*Eurotiales*). Front Microbiol 9:3058. (doi:10.3389/fmicb.2018.03058. 30619145 PMC6300479

[22] Fraaije B, Atkins S, Hanley S, Macdonald A, Lucas J. 2020. The multi-fungicide resistance status of *Aspergillus fumigatus* populations in arable soils and the wider European environment. Front Microbiol 11:599233. doi:10.3389/fmicb.2020.599233. 33384673 PMC7770239

[23] Kousha M, Tadi R, Soubani AO. 2011. Pulmonary aspergillosis: a clinical review. Eur Respir Rev 20:156–174. doi:10.1183/09059180.00001011. 21881144 PMC9584108

[24] Anagnostakis SL. 1987. Chestnut blight: the classical problem of an introduced pathogen. Mycologia 79:23–37. doi:10.2307/3807741.

[25] Smith JA, O’Donnell K, Mount LL, Shin K, Peacock K, Trulock A, Spector T, Cruse-Sanders J, Determann R. 2011. A novel *Fusarium* species causes a canker disease of the critically endangered conifer, *Torreya taxifolia*. Plant Dis 95:633–639. doi:10.1094/PDIS-10-10-0703. 30731893

[26] Weldon C, Du Preez LH, Hyatt AD, Muller R, Spears R. 2004. Origin of the amphibian chytrid fungus. Emerg Infect Dis 10:2100–2105. doi:10.3201/eid1012.030804. 15663845 PMC3323396

[27] USDA APHIS. 2012. 7 CFR Part 319 - Importation of wooden handicrafts from China. Federal Register 77(41):12437–12443.

[28] Kustrzeba-Wójcicka I, Siwak E, Terlecki G, Wolańczyk-Mędrala A, Mędrala W. 2014. *Alternaria alternata* and its allergens: a comprehensive review. Clin Rev Allergy Immunol 47:354–365. doi:10.1007/s12016-014-8447-6. 25205364

[29] Amaike S, Keller NP. 2011. Aspergillus flavus. Annu Rev Phytopathol 49:107–133. doi:10.1146/annurev-phyto-072910-095221. 21513456

[30] Hedayati MT, Pasqualotto AC, Warn PA, Bowyer P, Denning DW. 2007. *Aspergillus flavus*: human pathogen, allergen and mycotoxin producer. Microbiology (Reading) 153:1677–1692. doi:10.1099/mic.0.2007/007641-0. 17526826

[31] Bhunjun CS, Dong Y, Jayawardena RS, Jeewon R, Phukhamsakda C, Bundhun D, Hyde KD, Sheng J. 2020. A polyphasic approach to delineate species in *Bipolaris*. Fungal Diversity 102:225–256. doi:10.1007/s13225-020-00446-6.

[32] Zhu M, Fang Z, Zhuang M, Zhang Y, Lv H, Ji J, Yang L, Wang Y. 2021. First report of *Chaetomium globosum* causing leaf blight on *Brassica oleracea* L. in China. Plant Dis 105:1204–1204. doi:10.1094/PDIS-07-20-1540-PDN.

[33] Nam MH, Park MS, Kim HS, Kim TI, Kim HG. 2015. *Cladosporium cladosporioides* and *C. tenuissimum* cause blossom blight in strawberry in Korea. Mycobiology 43:354–359. doi:10.5941/MYCO.2015.43.3.354. 26539056 PMC4630446

[34] Anderson NR, Mehl KM, Neves DL, Bradley CA, Wise KA. 2019. First report of Curvularia leaf spot of corn, caused by *Curvularia lunata*, in Kentucky. Plant Dis 103:2692–2692. doi:10.1094/PDIS-03-19-0629-PDN.

[35] Heidarian R, Fotouhifar K-B, Debets AJM, Aanen DK. 2018. Phylogeny of *Paecilomyces*, the causal agent of pistachio and some other trees dieback disease in Iran. PLoS One 13:e0200794. doi:10.1371/journal.pone.0200794. 30040828 PMC6057626

[36] Sabernasab M, Jamali S, Marefat A, Abbasi S. 2019. Molecular and pathogenic characteristics of *Paecilomyces formosus*, a new causal agent of oak tree dieback in Iran. Forest Sci 65:743–750. doi:10.1093/forsci/fxz045.

[37] Gilbertson RL, Goldstein D, Lindsey JP. 1979. Additions to the check list and host index for Arizona wood-rotting fungi. J Arizona-Nevada Acad Sci 14:81–87. http://www.jstor.org/stable/40022228.

[38] Sarkar T, Chakraborty P, Karmakar A, Saha A, Saha D. 2019. First report of *Periconia macrospinosa* causing leaf necrosis of pointed gourd in India. J Plant Pathol 101:1281–1281. doi:10.1007/s42161-019-00348-w.

[39] Zhou H, Wang D, Zhao J, Dong B, Zhang X, Wen C, Zhang J. 2018. First report of *Rhizopus* head rot of sunflower caused by *Rhizopus arrhizus* (syn. *R. oryzae*) in Xinjiang and Gansu provinces of China. Plant Dis 102:1173–1173. doi:10.1094/PDIS-10-17-1528-PDN.

[40] Sinclair WA, Lyon HH. 2005. Diseases of trees and shrubs. Comstock Publishing Associates, Ithaca, NY.

[41] Chen J, Zhang L-C, Xing Y-M, Wang Y-Q, Xing X-K, Zhang D-W, Liang H-Q, Guo S-X. 2013. Diversity and taxonomy of endophytic xylariaceous fungi from medicinal plants of *Dendrobium* (*Orchidaceae*). PLoS One 8:e58268. doi:10.1371/journal.pone.0058268. 23472167 PMC3589337

[42] Ma H, Vasilyeva L, Li Y. 2013. The genus *Xylaria* (Xylariaceae) in the south of China-6: a new *Xylaria* species based on morphological and molecular characters. Phytotaxa 147:48–54. doi:10.11646/phytotaxa.147.2.2.

[43] Garrido-Arandia M, Silva-Navas J, Ramírez-Castillejo C, Cubells-Baeza N, Gómez-Casado C, Barber D, Pozo JC, Melendi PG, Pacios LF, Díaz-Perales A. 2016. Characterization of a flavonoid ligand of the fungal protein Alt a 1. Sci Rep 6:33468. doi:10.1038/srep33468. 27633190 PMC5025882

[44] Zhang D, Ge H, Xie D, Chen R, Zou H, Tao X, Dai J. 2013. Periconiasins A-C, new cytotoxic cytochalasans with an unprecedented 9/6/5 tricyclic ring system from endophytic fungus *Periconia* sp. Org Lett 15:1674–1677. doi:10.1021/ol400458n. 23506233

[45] Samarakoon BC, Wanasinghe DN, Samarakoon MC, Phookamsak R, McKenzie EHC, Chomnunti P, Hyde KD, Lumyong S, Karunarathna SC. 2020. Multi-gene phylogenetic evidence suggests *Dictyoarthrinium* belongs in *Didymosphaeriaceae* (*Pleosporales*, *Dothideomycetes*) and *Dictyoarthrinium musae* sp. nov. on *Musa* from Thailand. MycoKeys 71:101–118. doi:10.3897/mycokeys.71.55493. 32855605 PMC7423779

[46] Heshmatnia J, Marjani M, Mahdaviani SA, Adimi P, Pourabdollah M, Tabarsi P, Mahdavi F, Jamaati H, Adcock IM, Garssen J, Velayati A, Mansouri D, Mortaz E. 2017. *Paecilomyces formosus* infection in an adult patient with undiagnosed chronic granulomatous disease. J Clin Immunol 37:342–346. doi:10.1007/s10875-017-0395-5. 28429104

[47] Hattab Z, Ben Lasfar N, Abid M, Bellazreg F, Fathallah A, Hachfi W, Letaief A. 2019. *Alternaria alternata* infection causing rhinosinusitis and orbital involvement in an immunocompetent patient. New Microbes New Infect 32:100561. doi:10.1016/j.nmni.2019.100561. 31737277 PMC6849408

[48] Pastor FJ, Guarro J. 2008. Alternaria infections: laboratory diagnosis and relevant clinical features. Clin Microbiol Infect 14:734–746. doi:10.1111/j.1469-0691.2008.02024.x. 18727797

[49] Revankar SG, Sutton DA. 2010. Melanized fungi in human disease. Clin Microbiol Rev 23:884–928. doi:10.1128/CMR.00019-10. 20930077 PMC2952981

[50] Hafez N, Abdel-Razek AS, Hafez MB. 1997. Accumulation of some heavy metals on *Aspergillus flavus*. J Chem Technol Biotechnol 68:19–22. doi:10.1002/(SICI)1097-4660(199701)68:1<19::AID-JCTB508>3.0.CO;2-K.

[51] Siqueira JPZ, Sutton DA, Gené J, García D, Wiederhold N, Guarro J. 2018. Species of *Aspergillus* section *Aspergillus* from clinical samples in the United States. Med Mycol 56:541–550. doi:10.1093/mmy/myx085. 29420803

[52] Kim DM, Lee MH, Suh MK, Ha GY, Kim H, Choi JS. 2013. Onychomycosis caused by *Chaetomium globosum*. Ann Dermatol 25:232–236. doi:10.5021/ad.2013.25.2.232. 23717019 PMC3662921

[53] Shi D, Lu G, Mei H, de Hoog GS, Zheng H, Liang G, Shen Y, Li T, Liu W. 2016. Onychomycosis due to *Chaetomium globosum* with yellowish black discoloration and periungual inflammation. Med Mycol Case Rep 13:12–16. doi:10.1016/j.mmcr.2016.09.001. 27699147 PMC5035348

[54] Ma X, Hu J, Yu Y, Wang C, Gu Y, Cao S, Huang X, Wen Y, Zhao Q, Wu R, Zuo Z, Deng J, Ren Z, Yu S, Shen L, Zhong Z, Peng G. 2021. Assessment of the pulmonary adaptive immune response to *Cladosporium cladosporioides* infection using an experimental mouse model. Sci Rep 11:909. doi:10.1038/s41598-020-79642-y. 33441700 PMC7806624

[55] Lu X, Wang X, Zhang L, Li X, Qi X. 2020. Rare fungal keratitis caused by *Coprinellus radians*. Mycopathologia 185:389–394. doi:10.1007/s11046-019-00414-y. 31915988

[56] Carter E, Boudreaux C. 2004. Fatal cerebral phaeohyphomycosis due to *Curvularia lunata* in an immunocompetent patient. J Clin Microbiol 42:5419–5423. doi:10.1128/JCM.42.11.5419-5423.2004. 15528761 PMC525239

[57] Gunasekaran R, Janakiraman D, Rajapandian SGK, Appavu SP, Namperumalsamy Venkatesh P, Prajna L. 2021. *Periconia* species: an unusual fungal pathogen causing mycotic keratitis. Indian J Med Microbiol 39:36–40. doi:10.1016/j.ijmmb.2020.10.006. 33610254

[58] Bruni GO, Zhong K, Lee SC, Wang P. 2019. CRISPR-Cas9 induces point mutation in the mucormycosis fungus *Rhizopus delemar*. Fungal Genet Biol 124:1–7. doi:10.1016/j.fgb.2018.12.002. 30562583 PMC6784326

[59] Liu M, Bruni GO, Taylor CM, Zhang Z, Wang P. 2018. Comparative genome-wide analysis of extracellular small RNAs from the mucormycosis pathogen *Rhizopus delemar*. Sci Rep 8:5243. doi:10.1038/s41598-018-23611-z. 29588481 PMC5869740

[60] Sephton Clark P, Muñoz JF, Ballou E, Cuomo C, Voelz K. 2018. Pathways of pathogenicity: transcriptional stages of germination in the fatal fungal pathogen *Rhizopus delemar*. mSphere 3:00403-18.doi:10.1128/mSphere.00403-18. PMC6158513 30258038

[61] Gardiner BJ, Simpson I, Khuu MH, Kidd SE, Lo CH, Jenkin GA. 2015. An unusual ulcer: a case of cutaneous mucormycosis caused by *Rhizopus oryzae*. Med Mycol Case Rep 7:8–11. doi:10.1016/j.mmcr.2014.11.003. 27330940 PMC4909842

[62] Mohammadi R, Nazeri M, Sayedayn SMA, Ehteram H. 2014. A successful treatment of rhinocerebral mucormycosis due to *Rhizopus oryzae*. J Res Med Sci 19:72–74. 24672569 PMC3963327

[63] Rodríguez MM, Serena C, Mariné M, Pastor FJ, Guarro J. 2008. Posaconazole combined with amphotericin B, an effective therapy for a murine disseminated infection caused by *Rhizopus oryzae*. Antimicrob Agents Chemother 52:3786–3788. doi:10.1128/AAC.00628-08. 18694953 PMC2565887

[64] Rodríguez-Lobato E, Ramírez-Hobak L, Aquino-Matus JE, Ramírez-Hinojosa JP, Lozano-Fernández VH, Xicohtencatl-Cortes J, Hernández-Castro R, Arenas R. 2017. Primary cutaneous mucormycosis caused by *Rhizopus oryzae*: a case report and review of literature. Mycopathologia 182:387–392. doi:10.1007/s11046-016-0084-6. 27807669

[65] Cavanna C, Pagella F, Esposto MC, Tamarozzi F, Clemente L, Marone P, Matti E, Lallitto F. 2019. Human infections due to *Schizophyllum commune*: case report and review of the literature. J Mycol Med 29:365–371. doi:10.1016/j.mycmed.2019.100897. 31543381

[66] Kuhls K, Lieckfeldt E, Fau - Börner T, Börner T, Fau - Guého E, Guého E. 1999. Molecular reidentification of human pathogenic *Trichoderma* isolates as *Trichoderma longibrachiatum* and *Trichoderma citrinoviride*. Med Mycol 37:25–33. doi:10.1080/02681219980000041. 10200931

[67] Sandoval-Denis M, Sutton DA, Cano-Lira JF, Gené J, Fothergill AW, Wiederhold NP, Guarro J. 2014. Phylogeny of the clinically relevant species of the emerging fungus *Trichoderma* and their antifungal susceptibilities. J Clin Microbiol 52:2112–2125. doi:10.1128/JCM.00429-14. 24719448 PMC4042759

[68] Sugiyama K, Sano A, Murakami M, Ogawa T, Mishima H, Otake H, Kamei K, Sugiyama S. 2008. Three isolations of *Chaetomium globosum* from erythematous epilation of canine skin. Med Mycol 46:505–510. doi:10.1080/13693780801968555. 18608918

[69] Poutahidis T, Angelopoulou K, Karamanavi E, Karamanavi E, Polizopoulou ZS, Doulberis M, Doulberis M, Latsari M, Kaldrymidou E. 2009. Mycotic encephalitis and nephritis in a dog due to infection with *Cladosporium cladosporioides*. J Comp Pathol 140:59–63. doi:10.1016/j.jcpa.2008.09.002. 19064269

[70] El-Mougith AA. 1993. The effect of salinity on some halophilic soil fungi, p 473–477. *In* Lieth H, Al Masoom AA (ed), Towards the rational use of high salinity tolerant plants: deliberations about high salinity tolerant plants and ecosystems. Springer, Dordrecht, Netherlands.

[71] Jaouani A, Neifar M, Prigione V, Ayari A, Sbissi I, Ben Amor S, Ben Tekaya S, Varese GC, Cherif A, Gtari M. 2014. Diversity and enzymatic profiling of halotolerant micromycetes from Sebkha El Melah, a Saharan salt flat in southern Tunisia. BioMed Res Int 2014:439197. doi:10.1155/2014/439197. 25136587 PMC4124809

[72] Gaind S, Singh S. 2015. Production, purification and characterization of neutral phytase from thermotolerant *Aspergillus flavus* ITCC 6720. Int Biodeterioration Biodegradation 99:15–22. doi:10.1016/j.ibiod.2014.12.013.

[73] Nazareth S, Gonsalves V, Nayak S. 2012. A first record of obligate halophilic aspergilli from the dead sea. Indian J Microbiol 52:22–27. doi:10.1007/s12088-011-0225-z. 23449273 PMC3298590

[74] Nazir N, Mirza JH, Akhtar N, Bajwa R, Nasim G. 2007. Some studies of thermophilic and thermotolerant fungi from Lahore, Pakistan. Mycopathologia 5:95–100.

[75] Zámocký M, Tafer H, Chovanová K, Lopandic K, Kamlárová A, Obinger C. 2016. Genome sequence of the filamentous soil fungus *Chaetomium cochlioides* reveals abundance of genes for heme enzymes from all peroxidase and catalase superfamilies. BMC Genomics 17:763. doi:10.1186/s12864-016-3111-6. 27681232 PMC5041501

[76] Shao Z, Sun F. 2007. Intracellular sequestration of manganese and phosphorus in a metal-resistant fungus *Cladosporium cladosporioides* from deep-sea sediment. Extremophiles 11:435–443. doi:10.1007/s00792-006-0051-0. 17265162

[77] Zhang W, Yang R, Fang W, Yan L, Lu J, Sheng J, Lv J. 2016. Characterization of thermophilic fungal community associated with pile fermentation of Pu-erh tea. Int J Food Microbiol 227:29–33. doi:10.1016/j.ijfoodmicro.2016.03.025. 27046629

[78] De Faria FP, Te’O VSJ, Bergquist PL, Azevedo MO, Nevalainen KMH. 2002. Expression and processing of a major xylanase (XYN2) from the thermophilic fungus *Humicola grisea* var. thermoidea in *Trichoderma reesei*. Lett Appl Microbiol 34:119–123. doi:10.1046/j.1472-765x.2002.01057.x. 11849507

[79] Kaerger K, Schwartze VU, Dolatabadi S, Nyilasi I, Kovács SA, Binder U, Papp T, Hoog S.d, Jacobsen ID, Voigt K. 2015. Adaptation to thermotolerance in *Rhizopus* coincides with virulence as revealed by avian and invertebrate infection models, phylogeny, and physiological and metabolic flexibility. Virulence 6:395–403. doi:10.1080/21505594.2015.1029219. 26065324 PMC4604701

[80] Di Piazza S, Houbraken J, Meijer M, Cecchi G, Kraak B, Rosa E, Zotti M. 2020. Thermotolerant and thermophilic mycobiota in different steps of compost maturation. Microorganisms 8:880. doi:10.3390/microorganisms8060880. PMC7355412 32545162

[81] Peterson SW, Željko J. 2017. New species of *Talaromyces* isolated from maize, indoor air, and other substrates. Mycologia 109:537–556. doi:10.1080/00275514.2017.1369339. 29020573

[82] Shetaia YMH, El Khalik WAA, Mohamed TM, Farahat LA, ElMekawy A. 2016. Potential biodegradation of crude petroleum oil by newly isolated halotolerant microbial strains from polluted Red Sea area. Mar Pollut Bull 111:435–442. doi:10.1016/j.marpolbul.2016.02.035. 26902685

[83] Hosseyni Moghaddam MS, Safaie N, Soltani J, Hagh-Doust N. 2021. Desert-adapted fungal endophytes induce salinity and drought stress resistance in model crops. Plant Physiol Biochem 160:225–238. doi:10.1016/j.plaphy.2021.01.022. 33517220

[84] Kis-Papo T, Oren A, Fau - Wasser SP, Wasser Sp Fau - Nevo E, Nevo E. 2003. Survival of filamentous fungi in hypersaline Dead Sea water. Microb Ecol 45:183–190. doi:10.1007/s00248-002-3006-8. 12545316

[85] Bilal S, Khan AL, Shahzad R, Asaf S, Kang SM, Lee IJ. 2017. Endophytic *Paecilomyces formosus* LHL10 augments *Glycine max* L. adaptation to Ni-contamination through affecting endogenous phytohormones and oxidative stress. Front Plant Sci 8:870. doi:10.3389/fpls.2017.00870. 28611799 PMC5447229

[86] Konrad H, Stauffer C, Kirisits T, Halmschlager E. 2007. Phylogeographic variation among isolates of the *Sirococcus conigenus* P group. Forest Pathol 37:22–39. doi:10.1111/j.1439-0329.2007.00477.x.

[87] Koukol O, Kolařík M, Kolářová Z, Baldrian P. 2012. Diversity of foliar endophytes in wind-fallen *Picea abies* trees. Fungal Diversity 54:69–77. doi:10.1007/s13225-011-0112-2.

[88] Mitchell JK, Garrido-Benavent I, Quijada L, Pfister DH. 2021. Sareomycetes: more diverse than meets the eye. IMA Fungus 12:6. doi:10.1186/s43008-021-00056-0. 33726866 PMC7961326

[89] Gaber DA, Berthelot C, Camehl I, Kovács GM, Blaudez D, Franken P. 2020. Salt stress tolerance of dark septate endophytes is independent of melanin accumulation. Front Microbiol 11:562931. doi:10.3389/fmicb.2020.562931. 33362727 PMC7758464

[90] Bashiri S, Abdollahzadeh J, Di Lecce R, Alioto D, Górecki M, Pescitelli G, Masi M, Evidente A. 2020. Rabenchromenone and rabenzophenone, phytotoxic tetrasubstituted chromenone and hexasubstituted benzophenone constituents produced by the oak-decline-associated fungus *Fimetariella rabenhorstii*. J Nat Prod 83:447–452. doi:10.1021/acs.jnatprod.9b01017. 31967466 PMC7993755

[91] Tao M-h, Li D-l, Zhang WM, Tan JW, Wei XY. 2011. Study on the chemical constituents of endophytic fungus *Fimetariella rabenhorstii* isolated from *Aquilaria sinensis*. J Chin Med Mater 34:221–223. 21823477

[92] Mahish PK, Tiwari KL, Jadhav SK. 2015. Biodiversity of fungi from lead contaminated industrial waste water and tolerance of lead metal ion by dominant fungi. Res J Environ Sci 9:159–168. doi:10.3923/rjes.2015.159.168.

[93] Joshi PK, Swarup A, Maheshwari S, Kumar R, Singh N. 2011. Bioremediation of heavy metals in liquid media through fungi isolated from contaminated sources. Indian J Microbiol 51:482–487. doi:10.1007/s12088-011-0110-9. 23024411 PMC3209935

[94] Muthayya S, Sugimoto JD, Montgomery S, Maberly GF. 2014. An overview of global rice production, supply, trade, and consumption. Ann N Y Acad Sci 1324:7–14. doi:10.1111/nyas.12540. 25224455

[95] Manter DK, Vivanco JM. 2007. Use of the ITS primers, ITS1F and ITS4, to characterize fungal abundance and diversity in mixed-template samples by qPCR and length heterogeneity analysis. J Microbiol Methods 71:7–14. doi:10.1016/j.mimet.2007.06.016. 17683818

